# Parameter Estimation for Interrupted Sampling Repeater Jamming Based on ADMM

**DOI:** 10.3390/s21248277

**Published:** 2021-12-10

**Authors:** Chaoyu Wang, Wanwan Hu, Zhe Geng, Jindong Zhang, Daiyin Zhu

**Affiliations:** 1 Nanjing Marine Radar Institute, China Shipbuilding Industry Corporation, Nanjing 211100, China; wangchaoyv@outlook.com; 2 Key Laboratory of Radar Imaging and Microwave Photonics, Nanjing University of Aeronautics and Astronautics, Nanjing 211106, China; hww@nuaa.edu.cn (W.H.); zhegeng@nuaa.edu.cn (Z.G.); zjdjs@126.com (J.Z.)

**Keywords:** interrupted sampling repeater jamming (ISRJ), nonlinear integer optimization model, windowed vector, alternating direction method of multipliers (ADMM)

## Abstract

By repeatedly sampling, storing, and retransmitting parts of the radar signal, interrupted sampling repeater jamming (ISRJ) based on digital radio frequency memory (DRFM) can produce a train of secondary false targets symmetrical to the main false target, threatening to mislead or deceive the victim radar system. This paper proposes a computationally-effective method to estimating the parameters for ISRJ by resorting to the framework of alternating direction method of multipliers (ADMM). Firstly, the analytical form of pulse compression is derived. Then, for the purpose of estimating the parameters of ISRJ, the original problem is transformed into a nonlinear integer optimization model with respect to a window vector. On this basis, the ADMM is introduced to decompose the nonlinear integer optimization model into a series of sub-problems to estimate the width and number of ISRJ’s sample slices. Finally, the numerical simulation results show that, compared with the traditional time-frequency (TF) method, the proposed method exhibits much better performance in accuracy and stability.

## 1. Introduction

With the rapid development of radar-electronic warfare technology, the modern electronic countermeasure (ECM) is now capable of intercepting, analyzing, recognizing, and locating the radar signals of interest within a short period of time [1,2,3]. The emergence and utilization of digital radio frequency memory (DRFM) make it easier for the jammer to form a series of realistic coherent false targets, which are flexible, diverse, and highly resistant [2]. DRFM has two operating modes, i.e., full-pulse storage (FPS) mode and interrupted-sampling repeating (ISR) mode [4]. A jammer working in the FPS mode intercepts and retransmits the whole radar signal to generate the distributed false targets. Since the intercept-forward delay is longer than pulse duration, the largest signal processing gain is obtained in the FPS mode. A jammer operating in the ISR mode is termed as the interrupted sampling repeater jammer (ISRJ), which samples a slice of radar signal and retransmits it multiple times. The sample-retransmit process is repeated for several cycles until the falling edge of radar transmission signal is detected, so that a train of secondary false targets symmetrical with respect to the main false target is produced [5,6]. Compared with the traditional DRFM approaches, ISRJ is an intra-pulse jamming method with many advantages, which include receive-transmit time-sharing antenna, low-rate interrupted sub-sampling, flexible forwarding mode, and simultaneous deception and suppression effects [7,8,9,10].

Owing to its excellent performance, the ISRJ technique has been studied for more than a decade, and many researchers have made great contributions to the development and analysis of this jamming technique. In Zhou et al. [11], the overall framework of ISRJ was summarized and the key setting parameters, which define the number and quality of the false targets, were discussed. In Feng et al. [12], the mathematical principles of ISRJ against pulse-compression radar using linear frequency modulated (LFM) waveforms were studied, and the jamming effectiveness under wideband/narrowband conditions was discussed. In Li et al. [13], a modified strategy of ISRJ, combined with interception superposition and frequency shift modulation, was proposed for LFM radar. The intercepted slices were frequency modulated before being forwarded; thus the jamming could achieve better effects by increasing false targets and disordering their positions. In Li et al. [14], the relationship between slice width, forwarding times, and false target groups in the range direction was deduced, and the jamming effects with different jamming power were studied based on the coherent jamming principle and suppression mechanism. In addition, it should be stated that, similar to the LFM radar, ISRJ can also act against synthetic aperture radar (SAR) using nonlinear chirp waveforms [15,16,17,18]. However, considering the systematic advantages of quantum radar, ISRJ has not been involved in researching for it [19,20,21,22,23,24,25]. In brief, most achievements mentioned above focus on performance analysis and strategy modification for ISRJ based on the false target characteristics, such as amplitude, spatial distribution, and phase, etc.

Although the ISRJ has demonstrated outstanding performance in providing coherent repeater jamming for radar deception, there are actually some essential differences between the signal features of the ISRJ and the true targets [26,27,28,29]. However, there are few ISRJ classification approaches investigated in the published literature to extract the distinct features of ISRJ signal. 

In Zhou et al. [30], a sliding-truncation matched-filtering method was proposed, with which a two-dimensional search is employed in time delay and window width of the pulse compression (PC) to estimate the ISRJ signal width and the interval time. However, the limitation of this method is that the search range and search step should be known in advance. In Zhou et al. [31], the pulse compression results of ISRJ were analyzed by the time-frequency (TF) method to obtain the characteristic parameters, which include the number of sample slices and the period of forwarding. The slice width is then estimated further by deconvolution processing. However, this method is not applicable for the case that the jammer forwards multiple radar signals. In Zhan et al. [32], the short-time fractional Fourier transform (STFRFT) was applied to estimate the characteristic parameters of ISRJ, such as the sample duration and the forward interval. Nevertheless, the high computation cost of this method is troublesome. In Chen et al. [33], the neural network was employed to extract the distinct feature of the ISRJ signal, which offers a better recognition rate for ISRJ than that offered by the TF method. However, the training of the neural network requires massive real measured radar data and high computational load. In summary, most parameter estimation methods for ISRJ based on TF analysis have high computation complexity, which leads to obstacles for the real-time application in a practical radar system.

To investigate the intrinsic property of ISRJ effectively, this paper proposes a parameter estimation method with low computation cost by utilizing the alternating direction method of multipliers (ADMM) [34] to estimate the width and the number of sample slices. The main contributions of this work are summarized as follows:

(1) The parameter estimation of ISRJ is recast as a problem of windowed vector estimation from a new perspective, and a nonlinear integer optimization model is developed for parameter estimation to mitigate the high computational complexity suffered by brute two-dimensional TF analysis.
(2) The ADMM method is introduced to decomposing the nonlinear integer optimization problem into several simple sub-problems with lower computation complexity to estimate the width and the number of sample slices, which achieves better performance for ISRJ than the TF analysis methods used in the published literature.

The remaining contents of this paper are organized as follows: In Section 2, the analytical form of pulse compression results of ISRJ is deduced. In Section 3, the parameter estimation of ISRJ is transformed into a nonlinear integer optimization problem for windowed vector estimation. In Section 4, the nonlinear integer optimization model is decomposed into a discrete model and continuous model, and the proposed method is presented, including the framework and application of ADMM. Section 5 describes the numerical simulations for comparing the performance of the proposed method with TF based method in the published literature. Conclusions are drawn in Section 6.

## 2. ISRJ Signal Mode

**Figure 1:**
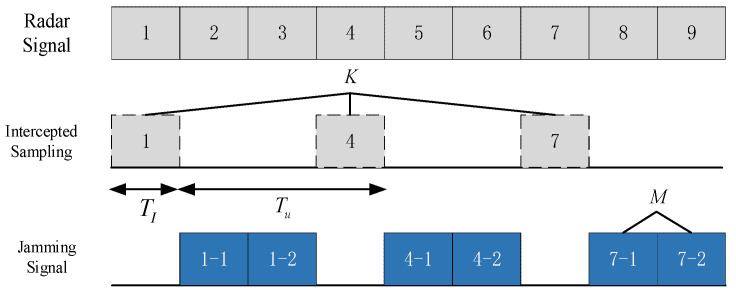
The jamming principle of ISRJ.

According to the generation mechanism of ISRJ, parts of radar signal are firstly intercepted and stored in the memory. Then, the jammer transmits the corresponding slice of signal repeatedly to counter the radar, which could generate a large number of more realistic coherent false targets [4]. The jamming principle of ISRJ can be illuminated as shown in Figure 1.

Assumed that the signal transmitted by radar can be expressed as [35,36]

(1) $$x\left(t\right)=\left\{ \begin{array}{l} \frac{1}{\sqrt{T_{p}}}e^{j2\pi f_{0}t}e^{j\pi K_{r}t^{2}}\;\left|t\right|\leq \frac{T_{p}}{2}\\ 0\;\left|t\right|>\frac{T_{p}}{2} \end{array}\right.$$

where $f_{0}$ is the carrier frequency of the radar signal, $T_{p}$ is the pulse width, $K_{r}=B/T_{p}$ is frequency modulation slope, and B is the signal bandwidth.

For ISRJ, a slice of the radar signal is intercepted and sampled by the jammer, which is able to generate the signal with the same carrier frequency and baseband as the radar transmission signal. Therefore, for the radar receiver, the ISRJ signal can be summarized as the intercept-forward radar baseband signal after digital down-conversion processing, which can be described as

(2) $$s_{I}\left(t\right)=\sum_{k=0}^{K-1}rect\left(\frac{t-\tau -kT_{u}}{T_{I}}\right)e^{j\pi K_{r}{\left(t-\tau \right)}^{2}}$$

where $T_{I}$ is the width of sample slice, K is the number of sampling slice, $T_{u}=(M+1)T_{I}$ is the interrupted-sampling interval, M is the times of each sample slice forwarded, τ is the forwarding delay and propagation delay of the sample slice, and rect(t/T)=1 for 0≤t≤T and zeros otherwise. It is worth noting that the amplitude components can be neglected since the term has no effect on the subsequent derivation of the jamming model.

If the same sample slice is forwarded M times, the Equation (2) can be rewritten as

(3) $$s_{J}\left(t\right)=\sum_{m=1}^{M}s_{I}\left(t-mT_{I}\right)=\sum_{m=1}^{M}\sum_{k=0}^{K-1}rect\left(\frac{t-\tau -kT_{u}-mT_{I}}{T_{I}}\right)e^{j\pi K_{r}{\left(t-\tau -mT_{I}\right)}^{2}}$$

In practice, ISRJ mainly includes direct repeating interference, repeatedly repeating interference, and cyclic repeating interference [26]. For convenience, the ISRJ only refers to direct repeating interference in this paper. It is assumed that the intercepted radar signal is forwarded with no delay until the falling edge of the radar signal. It is equivalent to set M=1 and τ=0, which means that the Equation (3) can be modified as

(4) $$s_{J}\left(t\right)=s_{I}\left(t-T_{I}\right)=\sum_{k=0}^{K-1}rect\left(\frac{t-T_{I}-kT_{u}}{T_{I}}\right)e^{j\pi K_{r}{\left(t-T_{I}\right)}^{2}}$$

Let $u_{k}\left(t-T_{I}\right)=\sum_{k=0}^{K-1}rect\left(\frac{t-T_{I}-kT_{u}}{T_{I}}\right)$, which represents the sum of K rectangular window functions. Therefore, for the Equation (4), ISRJ can be regarded as the radar transmitting signal x(t) with delayed $T_{I}$ weighted by the windows function $u_{k}\left(t-T_{I}\right)$.

In all, the Equation (4) could be rewritten as

(5) $$s_{J}\left(t\right)=u_{k}\left(t-T_{I}\right)e^{j\pi K_{r}{\left(t-T_{I}\right)}^{2}}$$

Let $t=nT_{s}$, $T_{I}=N_{I}T_{s}$ and $T_{u}=N_{u}T_{s}$, where $T_{s}$ is the sampling interval, $N_{I}$ is the number of sample points of the intercepted and stored radar signal and $N_{u}$ is the number of sample points of the repeat sampling interval. Thus, the Equation (5) can be described as

(6) $$s_{J}\left(n\right)=\sum_{k=0}^{K-1}rect\left(\frac{n-N_{I}-kN_{u}}{N_{I}}\right)e^{j\pi K_{r}T_{s}{}^{2}{\left(n-N_{I}\right)}^{2}}$$

For ISRJ, the pulse compression results can clearly represent the jamming capability of deception and suppression to radar. According to Equation (6), the pulse compression results of ISRJ can be expressed as

(7) $$\begin{array}{c} S_{MF}\left(n\right)=\sum_{k=0}^{K-1}\sum_{i=kN_{u}+\frac{N_{I}}{2}}^{kN_{u}+\frac{3}{2}N_{I}}e^{j\pi K_{r}T_{s}{}^{2}{\left(i-N_{I}\right)}^{2}}e^{-j\pi K_{r}T_{s}{}^{2}{\left(n-i\right)}^{2}}\\ \;=N_{I}T_{s}e^{j\phi }\sin c\left[K_{r}T_{s}{}^{2}N_{I}(n-N_{I})\right]\frac{1-e^{j2\pi K_{r}T_{s}{}^{2}\left(n-N_{I}\right)KN_{u}}}{1-e^{j2\pi K_{r}T_{s}{}^{2}\left(n-N_{I}\right)N_{u}}} \end{array}$$

where $\phi =2\pi K_{r}T_{s}{}^{2}N_{I}\left(n-N_{I}\right)+\pi K_{r}T_{s}{}^{2}\left(N_{I}{}^{2}-n^{2}\right)$.

The corresponding amplitude response of Equation (7) can be denoted as

(8) $$\left|S_{MF}\left(n\right)\right|=T_{s}N_{I}\left|\sin c\left[K_{r}T_{s}{}^{2}N_{I}(n-N_{I})\frac{\sin(K\delta )}{\sin(\delta )}\right]\right|$$

where $\delta =\pi K_{r}T_{s}^{2}N_{u}\left(n-N_{I}\right)$.

As seen from Equations (7) and (8), the spectrum of the pulse compression results of the sample slice appears on each discrete spectrum of the whole radar signal. The spectrum of the multiple jamming after superposition is equivalent to sample the main lobe of the spectrum of the whole radar signal. Additionally, the amplitude envelope of the ISRJ after pulse compression obeys the “sinc” function. And the critical parameters affecting the ISRJ efficiency are the number of sample slices K and the width of sample slices $T_{I}$, which can be estimated by the peak value along time dimension and frequency dimension of the pulse compression results of ISRJ, respectively. However, the above two parameters are related to the form of the window function $u\left(t-T_{I}\right)$. Hence, the estimation of $u\left(t-T_{I}\right)$ and $T_{I}$ can be transformed into estimating the window function.

## 3. Parameter Estimation Model

Suppose the unknown window function is q(n), the Equation (6) can be described as

(9) $$s_{J}\left(n\right)=\sum_{n=0}^{N-1}q\left(n-N_{I}\right)x\left(n-N_{I}\right)$$

where $q\left(n\right)=rect\left(\frac{n-kN_{u}}{N_{I}}\right)$, $x\left(n\right)=e^{j\pi K_{r}T_{s}{}^{2}n^{2}}$.

In addition, assuming that the echo of radar can be expressed as

(10) $$\left\{ \begin{array}{l} r_{1}\left(n\right)=\alpha_{t}s\left(n\right)+\alpha_{J}s_{J}\left(n\right)+w\left(n\right)\\ r_{0}\left(n\right)=\alpha_{J}s_{J}\left(n\right)+w\left(n\right) \end{array}\right.$$

where $r_{1}\left(n\right)$ represents the echo consisting of target echo s(n), ISRJ $s_{J}\left(n\right)$ and Gaussian white noise w(n), $r_{0}\left(n\right)$ represents the echo consisting of $s_{J}\left(n\right)$ and w(n), $\alpha_{t}$ is the coefficient of radar signal and $\alpha_{J}$ is the coefficient of jamming.

The difference between the pulse compression results of target echo and ISRJ can be written as

(11) $$\begin{array}{c} f\left[q\left(n\right)\right]=r_{0}\left(n\right)*s^{*}\left(-n\right)-r_{0}\left(n\right)*s_{J}^{*}\left(-n\right)\\ \;=\sum_{n=0}^{N-1}\left[s^{*}\left(-n\right)-q\left(-n\right)⊙s^{*}\left(-n\right)\right]*r_{0}\left(n\right) \end{array}$$

where * denotes convolution.

For Equation (11), the difference means the difference of the pulse compression results of the real window and estimated window functions of ISRJ. It means if the estimated window equals to the real window, the difference can be neglected, which should be 0 under the ideal conditions. However, in practice, the real window function is usually unknown. Hence, in order to measure the difference of Equation (11), the infinite norm is taken as follows;

(12) $${\left\|f\left[q\left(n\right)\right]\right\|}_{\infty }={\left\|Hq\right\|}_{\infty }$$

where H is the coefficient matrix, and q is the windowed vector.

According to Equation (12), when the minimum value of ${\left\|f\left[q\left(n\right)\right]\right\|}_{\infty }$ is obtained, the result of the radar signal with windows should be closest to the ISRJ signal. Therefore, the parameter estimation of ISRJ can be transformed into an optimization problem, realizing the estimation of the width and the number of sample slices for ISRJ. In order to obtain sparse solutions in the parameter estimation model and prevent overfitting, the regularization term is added in Equation (12). Therefore, the parameter estimation model for ISRJ is derived as

(13) $$\underset{q(n)}{\min}F[q\left(n\right)]={\left\|Hq\right\|}_{\infty }+{\left\|q\right\|}_{1}\;s.t.\;q\left(n\right)\in \{0,1\},\;n=1,2,\ldots N$$

where ${\left\|q\right\|}_{1}$ is the regularization.

It can be found that Equation (13) is about a nonlinear integer programming problem. The traditional methods to solve this type of problem mainly include the Branch-and-Bound (BB) method [37,38], generalized Benders decomposition (GBD) method, and so on. However, the BB method is a kind of traversal algorithm. When there are many integer variables in the problem, the BB method takes a long time, especially in large-scale optimization problems. GBD method usually decomposes the nonlinear integer programming problem into main and sub-problems to construct new constraints by duality theorem and finally transfer the optimal solution of the sub-problem to the solution of the main problem. But it should be noted that, in nonconvex problems, the GBD method cannot be guaranteed to accurately convey the solution of sub-problems to the main problem.

## 4. Parameter Estimation Method

### 4.1. ADMM Algorithm

The ADMM algorithm combines the advantages of the separability of the dual ascent method and the convergence of the multiplier method. Its essence is to decompose a massive problem into several small problems and solve them iteratively to make the original problem and the dual variables converge together [39].

The ADMM algorithm can be described as

(14) $$\left\{ \begin{array}{l} \min f(x)+g(z)\\ Ax+Bz=c \end{array}\right.$$

where f(x) and g(z) are both convex functions.

According to Equation (14), the Lagrangian function can be constructed as

(15) $$L_{p}(x,z,\lambda )=f(x)+g(z)+\lambda^{T}(Ax+Bz-c)+\frac{\rho }{2}{\left\|Ax+Bz-c\right\|}_{2}^{2}$$

where $L_{p}(x,z,\lambda )$ is the augmented Lagrangian function, λ is the dual variable, and ρ>0 is the penalty coefficient.

Based on Equation (15), the (k+1)th iteration of the ADMM algorithm can be expressed as

(16) $$\left\{ \begin{array}{l} x^{k+1}=\underset{x}{\arg\min}L_{p}(x,z^{k},\lambda^{k})\\ z^{k+1}=\underset{z}{\arg\min}L_{p}(x^{k+1},z,\lambda^{k})\\ \lambda^{k+1}=\lambda^{k}+\rho (Ax^{k+1}+Bz^{k+1}-c) \end{array}\right.$$

It should be noted that, in Equation (16), the ADMM algorithm is used to estimate x and z iteratively, and then to estimate λ. In the process of ADMM algorithm, x and z are estimated alternately, which are different from the two variables estimated simultaneously with augmented Lagrangian multiplier method. Therefore, the ADMM algorithm is more suitable for solving convex optimization problems with separable variables.

### 4.2. Parameter Estimation

For nonlinear integer optimization problems, the advantage of ADMM algorithm lies in that it can decompose them into integer problems and continuous problems to reduce the complexity of the optimization problem. Specifically, instead of integer variables, the algorithm substitutes the continuous variables with the same upper and lower bounds as those of the integer variables in the optimization of the continuous problems. Thus, Equation (13) can be rewritten as the following

(17) minF(p) s.t. q∈{0,1}, p∈[0,1]

(18) p−q=0

where p is a vector, in which elements are between 0 and 1.

When q can be changed continuously in (13), p and q can be obtained by Equation (17), which is constrained by the boundary in the Equation (18). It should be noted that the boundary denotes the relationship between p and q, ensuring that the optimal solution obtained can uniformly converge to q after p is used to optimize continuous problems instead of q. Besides, Equation (17) could be optimized in the form of the augmented Lagrangian penalty coefficient by introducing Lagrangian multiplier λ and penalty coefficient ρ, as shown in.

(19) $$L(p,q,\lambda )=F(p)+\lambda^{T}(p-q)+\frac{\rho }{2}{\left(p-q\right)}^{T}(p-q)$$

According to the iteration steps of ADMM, the model to be optimized is decomposed into two smaller sub-models. Equation (20) infers a nonlinear programming model (NLP) with p and Equation (21) illustrates the mixed-integer quadratic programming (MIQP) with q

(20) $$\left\{ \begin{array}{l} z_{1}=F(p)+(\lambda^{k}{)}^{T}(p-q^{k})+\frac{\rho }{2}{\left(p-q^{k}\right)}^{T}(p-q^{k})\\ s.t.\;p\in [0,1] \end{array}\right.$$

(21) $$\left\{ \begin{array}{l} z_{2}={\left(\lambda^{k}\right)}^{T}(p^{k}-q)+\frac{\rho }{2}{\left(p^{k}-q\right)}^{T}(p^{k}-q)\\ s.t.\;q\in \{0,1\} \end{array}\right.$$

where k represents iteration times.

The (k+1)th iteration of the proposed algorithm can be expressed as

(22) $$\left\{ \begin{array}{l} p^{k+1}=\underset{p}{\arg\min}F(p)+{\left(\lambda^{k}\right)}^{T}(p-q^{k})+\frac{\rho }{2}{\left(p-q^{k}\right)}^{T}(p-q^{k})\;\\ q^{k+1}=\underset{q}{\arg\min}{\left(\lambda^{k}\right)}^{T}(p^{k+1}-q)+\frac{\rho }{2}{\left(p^{k+1}-q\right)}^{T}(p^{k+1}-q)\\ \lambda^{k+1}=\lambda^{k}+\rho (p^{k+1}-q^{k+1}) \end{array}\right.$$

According to the convergence requirement of ADMM, when the residual ε, between $p^{k+1}$ and $q^{k+1}$ reaches the convergence accuracy $\varepsilon_{0}$, the iteration stops and the optimal solution $q^{k+1}$ is obtained, as shown in Equation Reference.

(23) $$\varepsilon ={\left\|p^{k+1}-p^{k+1}\right\|}_{2}<\varepsilon_{0}$$

where $\varepsilon_{0}$ is the threshold of the ending condition.

Summarizing, the basic flowchart of parameter estimation is shown below:

Step 1: Pulse compression is applied to the radar signal and ISRJ signal, respectively.
Step 2: The parameter estimation of ISRJ is transformed into a nonlinear integer optimization problem for windowed vector estimation.
Step 3: The nonlinear integer optimization model is decomposed into a discrete model and continuous model.
Step 4: The ADMM is used to estimate the width and number of sample slices for ISRJ.
Step 5: The width of sample slices and the number of sample slices are estimated iteratively until the residual error reaches convergence accuracy.

## 5. Simulation Results

**Figure 2:**
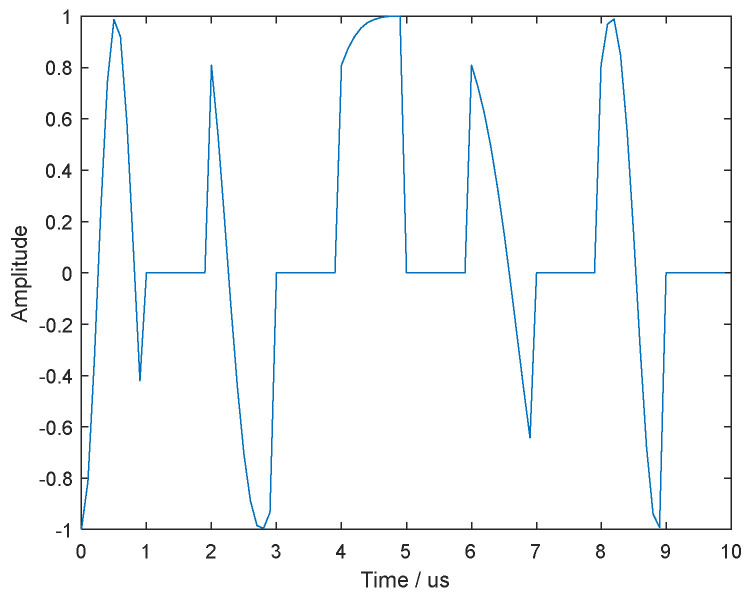
The time-domain waveform of ISRJ.

The simulation parameters are set as follows: the chirp signal is transmitted by radar, its bandwidth is B=2 MHz, the duration is τ=10 us, the sample frequency is $f_{s}=5B$, the jammer sample period is $T_{u}=0.2T$, the duty is r=0.5, the iteration ending threshold is $\varepsilon_{0}=0.001$, the background of the simulation is Gaussian white noise, and the jamming-to-noise ratio (JNR) is set to be 14 dB, which is defined as $JNR=10\mathrm{lg}\left(P_{J}/P_{N}\right)$, $P_{J}$ and $P_{N}$ denote the jamming power and the noise average power, respectively. Figure 2 shows the time-domain waveform of ISRJ.

**Figure 3:**
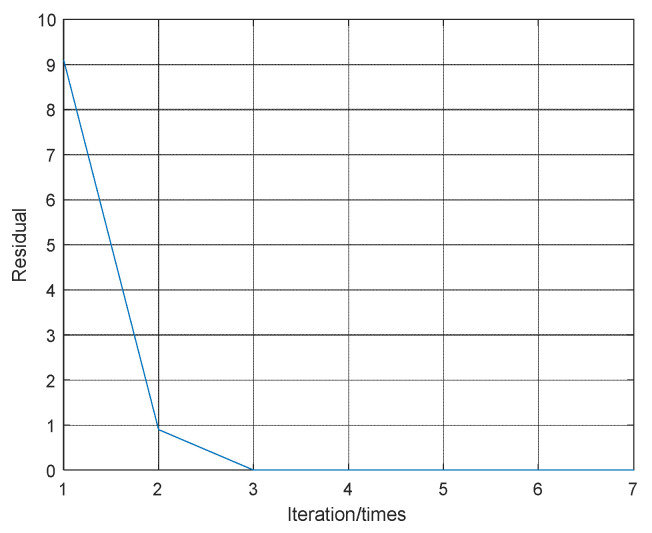
Residual convergence curve.

**Figure 4:**
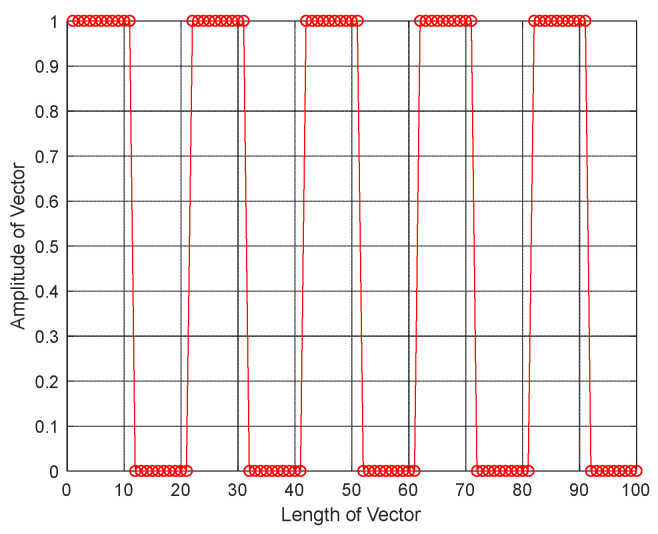
Estimated windowed vector.

As mentioned above, the residual is an important criterion for judging whether the ADMM algorithm is optimal. Figure 3 shows the residual convergence curve under JNR=14 dB, from which it can be concluded that the residual decreases as the number of iterations increases. Specifically, after three iterations, the residual ε approaches to zero, which indicates the proposed algorithm has superior to the convergence. Figure 4 is a diagram to show the estimation result of the windowed vector under the conditions mentioned above. It shows that the number and the width of sample slices can be estimated accurately.

**Figure 5:**
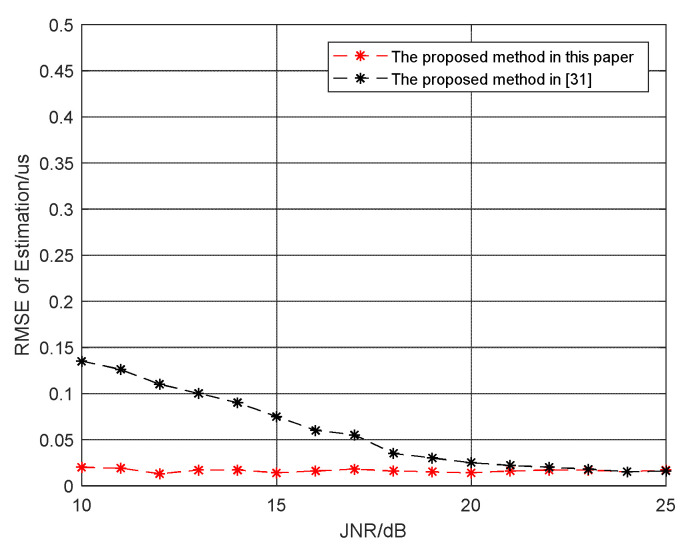
RMSE of slice width estimation.

In order to prove the effectiveness of the proposed method in this paper, 100 Monte Carlo simulation were carried out at JNR=10∼25 dB, and the root mean square error (RMSE) at each JNR was estimated and compared with the method proposed in Zhou et al. [31]. Figure 5 shows the RMSE of the two algorithms to estimate the width of the sample slice. It shows that the RMSEs of estimation for both methods decreases with the increase of JNR, especially when JNR > 10 dB, the RMSE of the proposed method coverage is to 0.02us. It should be noted that compared with the method in Zhou et al. [31], the estimation accuracy of the proposed method is greatly improved and is less affected by noise.

**Figure 6:**
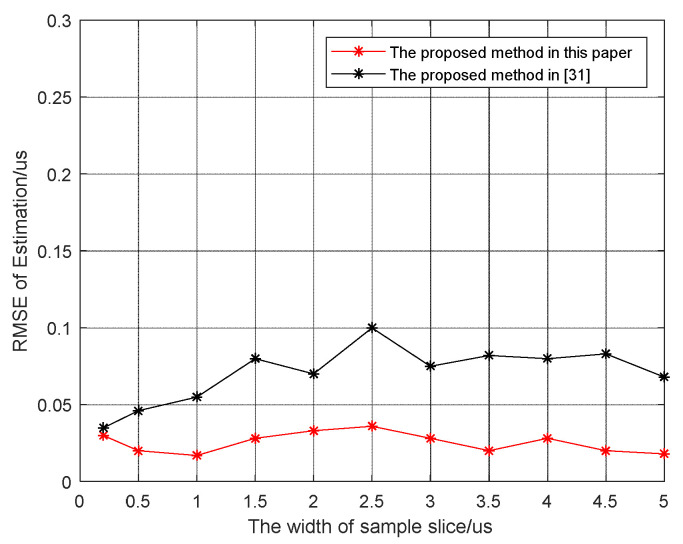
The root mean square error of estimated slice width varies with slice width.

In order to study the influence of the width of the sample slice on the proposed algorithm, 100 Monte Carlo simulations were performed at JNR=14 dB. Figure 6 shows the status of the estimation RMSE with respect to the width of the sample slice. It shows that the proposed algorithm was less affected by the width of the slice and the estimation RMSE is less than that of the method in Zhou et al. [31].

**Figure 7:**
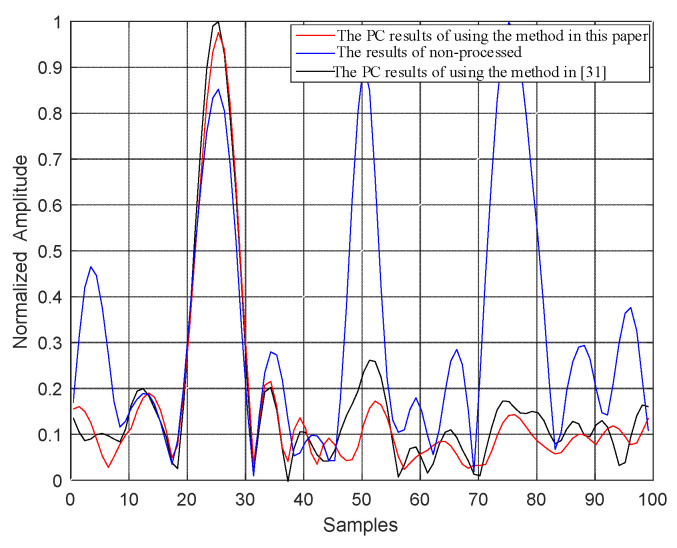
ISRJ suppressed results.

In order to test the jamming suppressing effectiveness based on the proposed algorithm, the radar echo is set as 0, and the ISRJ signal appears. In Figure 7, the blue line indicates the pulse compression results of radar echo, which exist three false targets. The red and black lines indicate the results of anti-ISRJ with the proposed method and the one presented in Zhou et al. [31], which first estimate the slice window of the ISRJ signal and then set the ISRJ signal as 0. The results indicate that the false targets generated from ISRJ can be suppressed successfully with the methods both of this paper and Zhou et al. [31]. In addition, since the proposed method can estimate the ISRJ signal more accurately than the method in [31], the sidelobe after pulse compression using the proposed method in this paper is lower than that of the result using the method in Zhou et al. [31].

**Figure 8:**
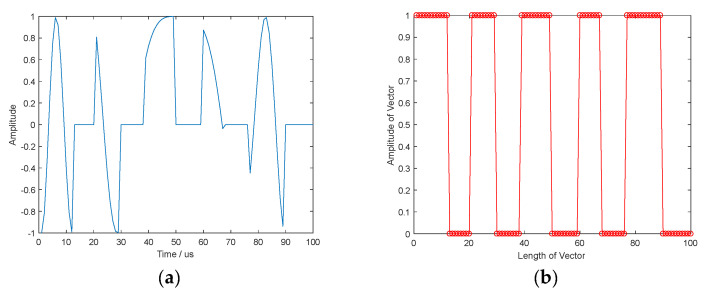
The time−domain waveform of ISRJ and estimated windowed vector with aperiodic train of rectangular pulses. (**a**) The time−domain waveform of ISRJ; (**b**) Estimated windowed vector.

In Figure 8, the proposed method based on ADMM is used to restore the widow function of aperiodic train of rectangular pulses. In Figure 8a, the time-domain waveform of ISRJ is plotted and shown in Figure 8b, the estimated window vector is given. The result indicates that the proposed method can obtain satisfactory restoration performance, which is similar to the case of the periodic train of rectangular pulses in Figure 4.

## 6. Conclusions

ISRJ is coherent with the radar signals and can produce a large number of realistic coherent false targets. For providing a premise and basis for the ISRJ countermeasure, this paper proposes a parameter estimation method based on ADMM. Based on the results of pulse compression for ISRJ, a nonlinear integer optimization model with a windowed vector is constructed. Furthermore, the framework of ADMM is introduced to estimate the windowed vector to obtain the width of the sample slice and the number of sample slices for ISRJ. The numerical simulations show that when the JNR ≥ 10 dB, compared with the time-frequency analysis method, the estimation accuracy of the parameters of the sample slice using the proposed method is significantly improved and more stable.

## Data Availability

Data sharing not applicable.
